# 

**Published:** 1849-12

**Authors:** 


### BOOKS RECEIVED.

We are indebted to the polite attention of Lea & Blanchard, of Philadelphia, for copies of the following books :

"The Three Kinds of Cod-Liver Oil; comparatively considered with reference to their chemical and therapeutic properties. By L. J. de Johngh, M. D., of the Hague. Translated from the German, with an appendix and cases, by Edward Carey, M. D."

"Surgical Anatomy. By Joseph Maclise, Surgeon, with colored plates."

We regret that these works did not reach us in time for a notice in our review department, but we cannot permit this number to go forth without urging upon the attention of students these valuable books. The treatise on cod-liver oil is of great value to the practitioner, and cannot fail to remunerate a close and diligent perusal.

The work of Maclise, on surgical anatomy, is of the highest value. In some respects it is the best publication of its kind we have seen, and is worthy of a place in the library of any medical man. It will be published in four parts, each one containing from twelve to sixteen colored plates. The whole work will make one large imperial quarto volume, with about one hundred and fifty double-columned pages, and from fifty to sixty magnificent plates, handsomely colored. The price of each part is two dollars, and a student could scarcely make a better investment than this.

These works are on sale at the bookstore of Beckwith & Morton.

B.

---

### THE CLOSE OF THE VOLUME.

We have been compelled to omit a number of articles prepared for this number, on account of the space occupied by the index. We have made it very full and complete, in order to give every possible facility to the reader, in finding the matter contained in the volume.

This number closes the present volume, and we hope to keep up a pleasant intercourse with at least the same readers, in the forthcoming volume. They may rely upon the fact, that no exertions shall be spared in making the Western Journal of Medicine and Surgery all that it should be. It shall be our constant object to advance the interests of the profession in all its departments, and to keep our readers fully up to the progress of medical science in all parts of the civilized world.

### INDEX TO VOL. IV.

### [THIRD SERIES.]

A
Abscesses, treated by nitrate of silver ....................162
Actual cautery in treatment of bloody tumors ....................308
Acute articular rheumatism—calomel in ....................437
Adulterated medicines ....................457
Alexandre & Co.'s new mechanical leech ....................254
American association for the advancement of science, proceedings of ....................275
Analogy and difference between tubercle and scrofula ....................255
Anatomy, by Quain ....................233
Anesthesia, or the employment of chloroform and ether in surgery, midwifery, &c ....................129
Anesthesia, Haskins on ....................291
Anesthesia from the local application of chloroform ....................435
Aneurism, femoral ....................260
Aneurism, Professor Dudley's paper on, reviewed ....................403
A new physiological fact ....................169
Annalist ....................368
Annan on cholera, reviewed ....................507
Annihilation of pains following surgical operations .................... 80
Antiphlogistic, arterial compression 259
Aphtha ....................251
Arsenic in cutaneous diseases ....................451
Arsenical preparations ....................378
Arterial compression as an antiphlogistic ....................259
Auscultation, manual of ....................336

B
Barbour, Professor, death of ....................89
Bartlett, Professor, character as a teacher ....................53
Barth & Rogers' manual of auscultation and percussion ....................336
Bauden's rules in treatment of fractures, gun-shot wounds, &c ....................539
Bell on practical medicine and surgery ....................369, 484
Bell on matico as an antihemorrhagic ....................376
Bell on arsenical preparations ....................378
Bell on neuralgic affections ....................380
Bell on croup ....................384
Bell on chloroform ....................385
Bell on collodion ....................400
Bevan's treatment of obstinate dyspepsia ....................523
Bird, his sulphur pills in cholera ....................178
Blackwell, Miss, an M. D. ....................366
Bladder, chronic inflammation of, treated with injections of nitrate of silver ....................77
Blair on intermittents and remittents ....................171
Blandin, Professor, death of ....................179
Bloodletting in pericarditis ....................432
Bloody tumors, treatment of ....................308
Books received ....................92, 184, 544
Brain, wound of ....................307
Brainard, Professor, on the use of collodion in the treatment of erectile tumors ....................448
Brainard on chloroform in periodical neuralgia ....................449
Bull on maternal management of children in health and disease ....................149

C
Calomel in acute articular rheumatism ....................437
Carroll on prevention of severe invasions of scarlatina ....................159

Cause of labor.................. 93
Cave, discovery of a new one in Kentucky....................364
Chambers on inhalation of nitrate of silver.....................166
Chilblains, collodion in............169
Children, maternal management of, in health and disease........149
Chloride of zinc, poisoning by....529
Chloroform, externally applied....366
Chloroform in parturition...... 384 84
Chloroform in periodical neuralgia.........................449
Cholera in Clarksville, Tenn...... 1
Cholera, progress of..........89, 180
Cholera, Scruggs on.............123
Cholera and other prevalent diseases.......................163
Cholera, Bird and his sulphur pills 178
Cholera in Rutherford county, Tenn........................269
Cholera, influenced by locality....271
Cholera and electricity ............273
Cholera, subsidence of.............363
Cholera infantum, use of coffee in.526
Cinchona, its commercial regulations and history................472
Cincinnati, mortality in............183
Clinical teaching, in the new amphitheater at the Louisville Marine Hospital...................274
Clinical reports of sterility........441
Cobb's case of facial palsy........469
Cod-liver oil in phthisis............ 83
Coffee in infantile cholera..........526
Colchicum in gonorrhea...........168
Cole's case of fracture of the skull 286
Collodion in chilblains.............169
Collodion in affections of the skin..400
Collodion in ulceration of the cervix uteri......................400
Collodion for chronic ulcers ......400
Collodion in laceration of the perineum........................401
Collodion, M. Ilisch's mode of preparing, and for vesication....401
Collodion, Livonius's mode of making, with great adhesive power..........................402
Collodion for the cure of erectile tumors.....................448
Compound fractures, amputations, bandage, &c.....................538
Cupping-glasses to the spine, for intermittent fever...............534
Cutaneous diseases, arsenic for....451

D

Death of Prof. Blandin............179
Death of eminent medical men....361
Death from one drop of laudanum.........................170
Delinquencies.....................368
Determinative cause of labor...... 93
Diagnosis of typhus and typhoid fever.........................245
Diarrhea and dysentery, spiritus pyroxylicus in..................185
Diarrhea, lienteric ................289
Discovery of a new cave in Kentucky.........................364
Disease of the heart, signs of, afforded by the hand laid over the precordia..................532
Dislocations of the elbow backwards, new method of reducing.........................162
Displacement of the uterus.......277
Dissertation on the practice of medicine......................334
Dropsy, congenital, case of....... 34
Dropsy after scarlet fever, treatment of........................534
Dudley, Professor, on aneurism, review of.....................403
Dudley's, Professor, surgical papers...........................455
Dysentery, epidemic...............460
Dyspeptic symptoms, treatment of.523

E

Ear, glycerine in diseases of.......348
Elbow joint, reduction of dislocation of.......................162
Elephant fossil....................363
Epidemic dysentery...............460
Erectile tumors, collodion in ......448
Errata...........................545

F

Fee's case of lienteric diarrhea....289
Fort's dissertation on the practice of medicine....................334
Fossil elephant....................363
Fracture of cranium—concussion and compression of the brain....488
Fracture of the skull, and perforation of the longitudinal sinus....286
Fracture of the neck of the femur within the capsule,—bony union 536
Fractures, compound, amputations, &c......................538
Froinus on vin. sem. colchici opiate in gonorrhea...............168
Functional derangement of the liver, associated with uterine derangement....................521

G

Gastro-intestinal affections, sub-nitrate of bismuth in.............438
Gonorrhea, colchicum in..........168
Gonorrhea, urethral pains following treatment of................168
Gordon on cause of labor ........ 93
Glycerine in diseases of the ear....348

H

Hargrave on opening cervical abscesses....................372
Hargrave's new operation for the ligature of the common carotid artery..........................375
Haskins on cholera................ 1
Haskins' cases of nephritis.........106
Haskins on medical heresies.......291
Hastings' work on surgery.........519
Heart disease, Yandell's case of....115
Heart, table of the pulses in diseases of the..................535
Hemorrhage, matico in.............376
Hepatic ducts, contents of, examined microscopically.............537
Hewett's report of a case of hydrophobia..........................202
History, natural, new work on....365
Hohl on prolapsus uteri........... 75
Hospital cases.......................110
Hospitals in Paris, statistics of.....175
Hospital report of cases, by Raphael...............................212
Human Anatomy, by J. Quain....233
Hydrarthrosis, nitrate of silver in...337
Hydrophobia, Hewett's report of a case...........................202

I

Inanition, prolonged................365
Intermittents and remittents, quinine in...............................171
Intermittent fever, treatment of, by the employment of cupping glasses to the spine.........534
Internal strangulation, strychnine in............................... 62
Iodide of potassium in chronic lead poisoning.....................527
Iodine, solution of, in oil.........524
Irregular malarious diseases, treatment of, by strychnine...........153

K

Kenney's case of wound of the brain—recovery....................307
Kentucky, discovery of a new cave in..............................364
Kirke's manual of physiology, review of...............221, 313, 413

L

Labor, cause of................... 93
Lane, on functional disease of the liver, associated with uterine derangement.......................521
Laudanum, death from one drop of..............................170
Lawrence's report of cases of puerperal peritonitis...........100
Lead Poison.........................458
Lead poison, chronic, iodide of potassium in......................527
Lead poison.........................458
Leech, mechanical..................254
Lee's report on sterility.............441
Lienteric diarrhea...................289
Lipscomb's case of congenital dropsy.......................... 34
Lipscomb on the use of the trephine in injuries of the head....461
Louisville University...............361
Lumbo-renal abscess...............106

M

MacDonnel on the treatment of chronic inflammation of the bladder by injections of nitrate of silver..................... 77
Maissonneuve's method of reducing dislocations of the elbow joint.........................162
Malarious diseases, strychnine in ..153
Manual of auscultation and percussion..............................336
Marchal on solution of iodine in oil.................................524
Matico, Bell on......................376
Mechanical leech...................254
Medical convention of Ohio.......177
Medical men, death of.............361
Medical reports, southern..........364
Medical Journal of Transylvania..367
Medical Journal, New Orleans....368
Medical department of Louisville University................453, 543
Memphis Medical College.........367
Mercury and bloodletting in pericarditis...........................432
Mesmerism in Holland............360
Microscopic examination of the contents of the hepatic ducts, with conclusions founded thereon................................537
Miller's, Professor, work on human parturition.................458
Monneret on sulphate of bismuth in gastro-intestinal affections.....438
Mortality in Cincinnati............183

N

Natural history, new work on.....365
Nephritis, cases of, by Yandell..... 15
Nephritis, Haskins' case of.........106
Neuralgia, periodical, use of chloroform in......................449
New Orleans Medical Journal.....368
Nitrate of silver in chronic inflammation of the bladder............ 77
Nitrate of silver in treatment of abscesses..........................162
Nitrate of silver, inhalation of.....166
Nitrate of silver as a vesicant......252
Nitrate of silver for white swellings, hydrothrosis, and venereal bubo..........................337

Nonat on treatment of abscesses by nitrate of silver....162
Nurse's sore mouth....251

O

Obstetrics, the science and the art...37
Obstinate dyspeptic symptoms, treatment of....523
Ohio legislature and quackery....368
Ohio Medical College.... 91
Ohio medical convention....177
On the neck as a medical region...339
Osborne's cases of thoracic disease 23
Ozone.... 87

P

Pancreatic juice, use of, in digestion.... 62
Paralysis, facial....469
Paralysis, paroxysmal....339
Paris, hospitals and medical profession in, statistics of....175
Parturition, chloroform in....384
Percussion and auscultation, manual of....336
Pericarditis, blood-letting and mercury in....432
Periodical neuralgia, chloroform in 449
Peritonitis puerperal, three cases of....100
Perspiration sanguineous....436
Phlebotomy in ancient times....437
Photophobia....247
Phthisis, cod-liver oil in.... 83
Phyllerine, sulphate of....360
Physiological fact, a new one....169
Physiology, manual of, review of....221, 313, 413
Poisoning by chloride of zinc....529
Practice of medicine, a dissertation on....334
Practical medicine and surgery....369
Proceedings of the American Association for the advancement of science....275
Professor of Materia Medica in the Louisville medical school.... 86
Prolonged inanition....365

Q

Quackery and the legislature of Ohio....368
Quain's anatomy....233
Quinine in West India remittent and intermittent fever....171

R

Raphael's hospital cases....110
Reduction of dislocation of elbow joint....162
Reflex nervous action of the uterus....430
Remittents, quinine in....171
Report of hospital cases by B. I. Raphael M. D....212
Reports, Southern Medical....364
Reviews—
Obstetrics, the science and the art. By Chas. D. Meigs, M. D.... 37
Anesthesia, or the employment of chloroform and ether in surgery, midwifery, etc. By J. T. Simpson, M. D., F. R. S., &c., &c....129
The Maternal management of children in health and disease. By Thomas Bull, M. D. 149
Manual of Physiology. By W. Senhouse Kirkes, M. D.... 221, 313, 413
Human Anatomy. By Jonas Quain, M. D....233
A Dissertation on the Practice of Medicine. By T. Fort, M. D..334
A Manual of Auscultation and Percussion. By M. Barth & M. Roger.... 336
Prof. Dudley on Aneurism....403
On the Diseases of the Bones. By E. Stanley, F. R. S., &c...493
Prof. Annan on Cholera....511
The Practice of Surgery, &c. By. John Hastings, M. D., U. S. N., &c....519
Rheumatism, acute articular, calomel in....437
Richmond Medical College....179
Rigidity of the os uteri, treatment..531
Rogers, Prof. Lewis, debut as a teacher....455
Roux on the annihilation of pains which follow surgical operations. 80

S

Sanguineous perspiration....436
Scarlatina, prevention of severe invasions of....159
Science, association for the advancement of, proceedings of...275
Scrofula and tubercle, analogies and differences....255
Scruggs on cholera....123
Scruggs' case of displacement of the uterus....277
Scull, fracture of, and perforation of longitudinal sinus....285
Short, Professor, resignation of.... 85
Signs of diseased heart afforded to the hand placed over the precordia....532
Silliman, Benj., Jr. Prof. as a lecturer....455
Silliman's, Prof, translation of review of Weddell's work on cinchona forests, &c....472
Simpson on anesthesia....129

Simultaneous development of variola and vaccinia................256
Smith, Dr W. Tyler, on reflex nervous action of the uterus.....430
Solution of iodine in oil...........524
Southern Medical Reports.........364
Spina bifidi cured by iodine........ 61
Spiritus pyroxylicus in diarrhea and dysentery....................185
Stanley on diseases of the bones, review of.......................511
Statistics of the hospitals and medcal profession in Paris..........175
Sterility, Lee's clinical reports on..441
Stone, Dr., on compound fractures, gun-shot wounds, amputations, &c.................................538
Strychnine in the treatment of internal strangulation of intestines.. 62
Strychnine in irregular malarious disease.............................153
Subnitrate of bismuth in gastrointestinal affections..............438
Subsidence of cholera.............363
Sugar in the animal economy, origin of.............................. 61
Sulphate of iron in gonorrhea and chancre..........................525
Surgery of cervical abscesses.....372
Surgical papers of Prof. Dudley...455
T
Table of the pulses in diseases of the heart..........................535
The heart-clot, its cause............ 63
The neck as a medical region.......339
Thoracic disease, cases of.......... 23
Transylvania University........... 92
Transylvania Journal of Medicine.367
Treatment of urethral pains.......168
Trephine, use of, in injuries of the head................................461
Tubercle and scrofula, analogies and differences.....................255
Typhus and typhoid fever, diagnosis of...........................245
U
University of Louisville...........361
Upshur on variola and vaccinia....429
Urethral pains, treatment of........168
Uric acid in the kidneys, a sign of a child having been born alive...530
Uterus, prolapsus of............... 75
Uterus, displacement of............277
Uteri os rigidity, treatment of......531
V
Variola and vaccinia, simultaneous development of...................256
Variola and vaccine.................429
Venereal bubo, use of nitrate of silver in,........................337
Vesication by nitrate of silver.....252
W
Walker's rules in the treatment of fractures, gunshot wounds, &c...541
White swelling, nitrate of silver for..............................337
Williams in cod-liver oil in phthisis. 83
Wooten's case of death from one drop of laudanum................170
Wound of the brain—recovery....307
Y
Yandell's cases of nephritis........ 15
Yandell's case of heart disease.....115
Yandell on spiritus pyroxylicus.....185
Yandell on treatment of bloody tumors by actual cautery........308
Z
Zymotic diseases....................245

### DELINQUENT SUBSCRIBERS.

This numerous class must see the necessity of making prompt remittances. We furnish a Journal which is not only a record of the progress of all the departments of Practical Medicine, but which contains the principles of practice adapted to the West and South. Its publication is attended with a heavy expenditure, and the subscribers should enable us to meet this expenditure without any trouble. We feel every assurance that we give them their money's worth, and we hope they will promptly respond to this call by remitting their dues.

PRENTICE & WEISSINGER, *Publishers.*

November 1st, 1849.

---

### *Receipts of Medical Journal for November*, 1849.

| | |
|---|---|
| Dr. —— Wheat, Edmonton, Ky. | $10 00 |
| J. C. Bernard, Haynesville, Mo. | 10 00 |
| E. H. Harris, Cape Girardeau, Mo. | 5 00 |
| F. Chilton, Genoa, Ky. | 5 00 |
| J. C. Harris, Wetumpka, Ala. | 10 00 |
| J. M. Stucky, Gosport, Ind. | 5 00 |
| S. B. Hobbs, Highland, Mo. | 10 00 |
| S. W. W. Brown, Leavenworth, Ind. | 10 00 |
| A. Clapp, New Albany, Ind. | 5 00 |
| —— Burrus, Memphis, Tenn. | 5 00 |

---

The WESTERN JOURNAL OF MEDICINE and SURGERY is published monthly by the undersigned, at the corner of Main and Fifth streets, Louisville, at $5 per annum, payable in advance. Each number contains 96 pages, making two volumes in the year of more than 550 pages each.

Contributions to its pages by the Physicians of the Valley of the Mississippi addressed tothe Editors, are respectfully solicited.

Letters on business, to be addressed, postage paid, to the publishers.

Jauuary, 1844. PRENTICE & WEISSINGER.

